# Subjective Cognitive Complaints: Heritability and Correlations with Dementia Diagnosis, Depression and Memory, Across Multiple Twin Studies

**DOI:** 10.1002/alz.088371

**Published:** 2025-01-03

**Authors:** Ashleigh S. Vella, Amanda Selwood, Vibeke S. Catts, Katya T. Numbers, Teresa Lee, Anbupalam Thalamuthu, Ida K Karlsson, Chandra A. Reynolds, Carol E. Franz, William S. Kremen, Margaret Gatz, Perminder S. Sachdev

**Affiliations:** ^1^ Centre for Healthy Brain Ageing, University of New South Wales, Sydney, NSW Australia; ^2^ Centre for Healthy Brain Ageing (CHeBA), UNSW Sydney, Sydney, NSW Australia; ^3^ Centre for Healthy Brain Ageing (CHeBA), University of New South Wales, UNSW Sydney, NSW Australia; ^4^ Karolinska Institutet, Stockholm Sweden; ^5^ University of California, Riverside, Riverside, CA USA; ^6^ University of California, San Diego, La Jolla, CA USA; ^7^ University of Southern California, Los Angeles, CA USA; ^8^ Neuropsychiatric Institute, Prince of Wales Hospital, Randwick, NSW Australia

## Abstract

**Background:**

Subjective Cognitive Complaints (SCCs) can often precede mild cognitive impairment and dementia longitudinally. While increasingly considered an early prodromal stage of dementia, SCCs can also be a symptom of depression. Previous research found that SCCs in the absence of cognitive impairment, controlling for symptoms of depression, were moderately heritable and genetically associated with memory. We aimed to replicate these previous findings by pooling data from a large consortium of twin studies across various countries.

**Method:**

Data containing 2,041 participants (902 twin pairs; 497 MZ pairs) were pooled from twin studies contributing to the Interplay of Genes and Environment across Multiple Studies (IGEMS) consortium (Older Australian Twin Study (OATS); Study of Origins of Variance in the Oldest‐Old (OCTO‐Twin); and Vietnam Era Twin Study of Aging (VETSA)). Univariate AE model investigated the heritability estimates using four variables (depression, SCCs, word‐list recall and prose recall) from OATS, OCTO‐Twin, and VETSA. Multivariate models, using VETSA and OATs, calculated the genetic correlations between harmonised SCC scores, memory, and depression. Fisher’s z‐transformed correlations were meta‐analysed to obtain the combined estimates of heritability and genetic correlations.

**Results:**

Consistent with previous research, SCC had a low combined heritability of 0.22(95% CI: [0.13,0.31]). The genetic influence on SCCs and memory were significantly related, such that as memory complaints increased memory performance decreased (word list recall, ‐0.17, 95% CI: [‐0.25, ‐0.10], prose recall, ‐0.21, 95% CI: [‐0.29, ‐0.14]), *p* < 0.05. An increase in depressive symptoms genetically correlated with a decline in memory (word list recall, ‐0.08, 95% CI: [‐0.16, ‐0.01], prose recall, ‐0.40, 95% CI: [‐0.49, ‐0.34]). No significant environmental correlations were observed between memory and either SCCs, or separately depression, *p* > 0.05.

**Conclusions:**

Consistent with our previous research, the heritability of SCC was low. The findings underscore the genetic connection between SCCs, objective memory performance, and depressive symptoms. The low heritability, however, suggests a substantial influence of environmental factors in explaining individual differences in SCC, highlighting the need for further research to better understand the dynamic influence of genetic and environmental influences on cognitive health.

